# A Draft Reference Genome Assembly of the Critically Endangered Black Abalone, *Haliotis cracherodii*

**DOI:** 10.1093/jhered/esac024

**Published:** 2022-05-14

**Authors:** Chloé Orland, Merly Escalona, Ruta Sahasrabudhe, Mohan P A Marimuthu, Oanh Nguyen, Eric Beraut, Blythe Marshman, James Moore, Peter Raimondi, Beth Shapiro

**Affiliations:** Ecology and Evolutionary Biology Department, University of California Santa Cruz, Santa Cruz, CA, USA; Department of Biomolecular Engineering, University of California Santa Cruz, Santa Cruz, CA, USA; UC Davis Genome Center, DNA Technologies and Expression Analysis Cores, University of California, Davis, Davis, CA, USA; UC Davis Genome Center, DNA Technologies and Expression Analysis Cores, University of California, Davis, Davis, CA, USA; UC Davis Genome Center, DNA Technologies and Expression Analysis Cores, University of California, Davis, Davis, CA, USA; Ecology and Evolutionary Biology Department, University of California Santa Cruz, Santa Cruz, CA, USA; Marine Laboratory, University of California Davis, Davis, CA, USA; Marine Laboratory, University of California Davis, Davis, CA, USA; Ecology and Evolutionary Biology Department, University of California Santa Cruz, Santa Cruz, CA, USA; Ecology and Evolutionary Biology Department, University of California Santa Cruz, Santa Cruz, CA, USA; Howard Hughes Medical Institute, University of California Santa Cruz, Santa Cruz, CA, USA

**Keywords:** black abalone, genetic diversity, long-read assembly, scaffolded assembly, whole genome

## Abstract

The once abundant black abalone, *Haliotis cracherodii*, is a large, long-lived grazing marine mollusk that inhabits the rocky intertidal along the coast of California. The species has experienced dramatic declines since the mid-1980s largely due to the fatal bacterial disease called withering syndrome, leading to the collapse of an economically important fishery and to its inclusion into the IUCN listing as a critically endangered species. In some places impacted by the disease, populations of black abalone have declined by more than 90%, prompting population crashes associated with very little recruitment of new individuals and changes to intertidal communities. Habitats that were dominated by crustose coralline algae and bare rock have become dominated instead by fleshy algae and sessile invertebrates. Here, we present the first high-quality black abalone reference genome, assembled with PacBio HiFi long-reads and assembled with Dovetail Omni-C data to generate a scaffold-level assembly. The black abalone reference genome will be an essential resource in understanding the evolutionary history of this species as well as for exploring its current levels of genetic diversity and establishing future management and restoration plans.

Black abalone (*Haliotis cracherodii*, Leach 1814) are large, long-lived gastropods typically found in the mid and low zones in rocky intertidal habitats and less often subtidally to a depth of 6 m (Morris et al. 1980). Their current range along the Pacific coast extends from Point Arena, in Northern California, USA, to Bahia Tortugas and Isla Guadalupe, in Southern Baja California, Mexico. Adult black abalone play a key role in maintaining favorable habitat for conspecific recruitment on rocky intertidal reefs, by facilitating encrusting coralline algae and influencing community structure (Cox 1962; Richards and Davis 1993; Miner et al. 2006). They are dioecious and reproduce by broadcast spawning, and their diet consists primarily of drift brown algae like feather boa and giant kelp (Leighton and Boolootian 1963; Morris et al. 1980; Blecha et al. 1992).

While they were once exceptionally abundant in California, they are now a threatened and endangered marine invertebrate. Losses of 90–99% of their population are the result of overfishing, environmental changes such as oil spills, sea temperature rise, and landslide sediment burials, and diseases including withering syndrome (WS) (Morris et al. 1980; VanBlaricom 1993; VanBlaricom et al. 2009). Among the seven abalone species found along the California coast, black abalone have been the most radically affected by WS (Lafferty et al. 1993; Raimondi et al. 2002; Miner et al. 2006). WS is caused by the bacterium *Candidatus Xenohaliotis californiensis* which attacks the lining of the digestive tract and results in reduced body mass, weakness, and eventual withering of the abalone’s foot until it can no longer cling to the substratum (Friedman et al. 2000). These dramatic declines have led to the closure of a 150-year-old economically important fishery in 1993, as well as to substantial changes to the intertidal ecosystem, with habitats that were dominated by crustose coralline algae and bare rock now dominated instead by fleshy algae and sessile invertebrates (Miner et al. 2006).

Consequently, black abalone are listed as Critically Endangered on the Red List of Endangered Species of the International Union for the Conservation of Nature (Smith et al. 2003) and have been protected in the United States under the Endangered Species Act since 2009. WS affected coastal populations differently though, with individuals remaining healthy in Central California but heavily affected south of Point Conception. Since the 2000s, black abalone have reappeared in some southern locations. These observations have prompted scientists and managers to work toward recovery actions such as translocations from one region to another or captive breeding. However, unlike for the white abalone (Rogers-Bennett et al. 2016), black abalone have not yet been successfully bred in captivity, meaning that restoration efforts will most likely rely on outplanting from wild populations.

Research suggests that because of their large, negatively buoyant gametes (Hamm and Burton 2000) and their shorter breeding season which coincides with limited oceanographic conditions (Leighton 1974), black abalone dispersal may be lower than that of other abalone species (Withler 2000; De Wit and Palumbi 2013; Gruenthal et al. 2014) and their populations more structured (Chambers et al. 2006). With only a few scattered individuals remaining, this restricted gene flow may be greater than previously imagined and may lead to low genetic diversity within populations (i.e., potential inbreeding) and to high genetic divergence among populations (i.e., metapopulations), which would complicate their translocation. A reference genome is now imperative if we wish to broaden our understanding of black abalone population genetics.

Here, we present the first high-quality de novo assembly for the black abalone genome. We generated the data from a captive black abalone using PacBio HiFi long-reads and Omni-C data. Our final genome spans 1.18 Gb across 82 scaffolds, with a scaffold N50 of 60Mb and a BUSCO complete score of 97.4%. This high-quality reference genome (37× coverage) will enable us to explore the evolutionary history of black abalone and will facilitate future research on their genetic structure and connectivity.

## Methods

### Biological Materials

**Figure 1.**
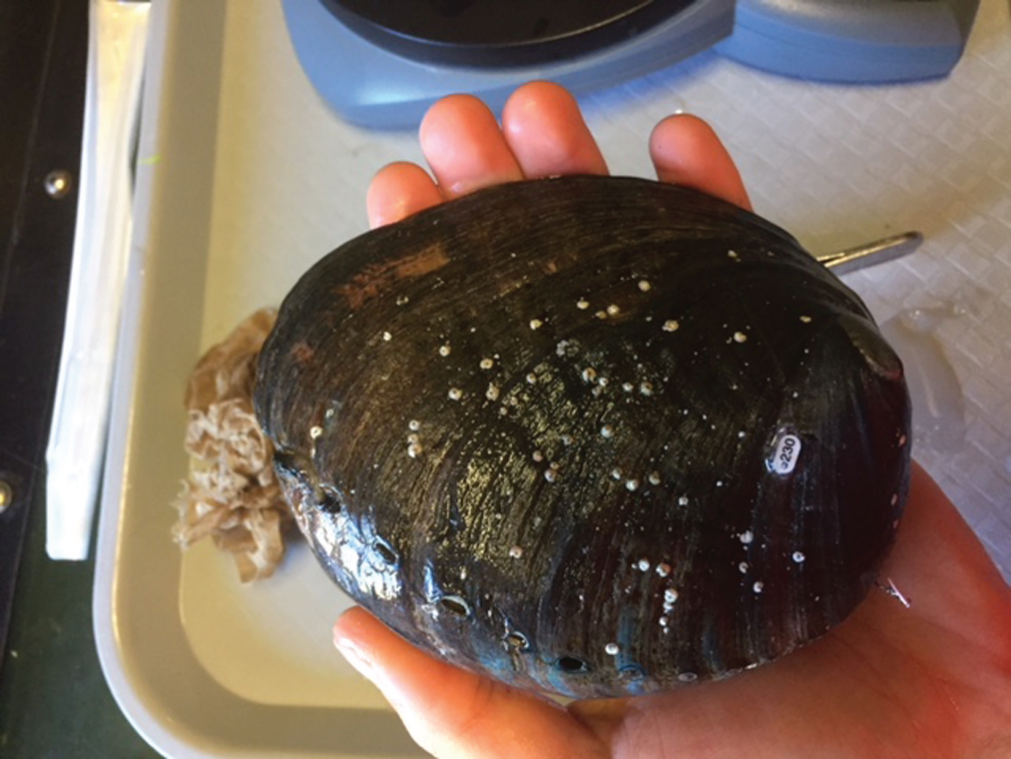
*Haliotis cracherodii*, black abalone, specimen used for the reference genome. Photo taken by Blythe Marshman.

The black abalone (W230) from which the tissue sample was provided was part of the captive population housed at the California Department of Fish and Wildlife’s shellfish pathology lab at UC Davis’ Bodega Marine Laboratory (Figure 1). A live tissue sample from the animal’s epipodia was collected on 21 July 2020 for this project under the federal black abalone permit 19571. This individual was originally collected in the wild by Carolyn Friedman in Carmel, CA, in 2005, and kept in captivity at the University of Washington for 9 years before being sent to UC Davis. The sex of the animal could not be determined visually or via histology as not enough gonad was visible, possibly due to the specimen’s age.

### Nucleic Acid Library Preparation and Sequencing

#### Nucleic Acid Extractions

High molecular weight (HMW) **genomic** DNA **(gDNA)** was extracted from 97 mg of epipodial clippings (Sample#TAG_230_A) using Nanobind Tissue Big DNA kit (Circulomics, Baltimore, MD) following the manufacturer’s instructions with minor modifications. The purity of the DNA was assessed on a NanoDrop spectrophotometer (260/280 = 1.77 and 260/230 = 1.92). DNA yield (125 ng/μL; 23 μg total) was quantified using Quantus Fluorometer (QuantiFluor ONE dsDNA Dye assay, Promega, Madison, WI; cat. E6150). The integrity of the HMW gDNA was estimated using the Femto Pulse system (Agilent Technologies, Santa Clara, CA), where 69% of the DNA fragments were found to be >100 Kb.

#### Pacific Biosciences HiFi Library

The HiFi SMRTbell library was constructed using the SMRTbell Express Template Prep Kit v2.0 (Pacific Biosciences—PacBio, Menlo Park, CA, Cat. #100-938-900) according to the manufacturer’s instructions. HMW gDNA was sheared to a target DNA size distribution between 15 kb and 20 kb. The sheared gDNA was concentrated using 0.45× of AMPure PB beads (PacBio, Cat. #100-265-900) for the removal of single-strand overhangs at 37 °C for 15 min, followed by further enzymatic steps of DNA damage repair at 37 °C for 30 min, end repair and A-tailing at 20 °C for 10 min and 65 °C for 30 min, ligation of overhang adapter v3 at 20 °C for 60 min and 65 °C for 10 min to inactivate the ligase, then nuclease treated at 37 °C for 1 h. The SMRTbell library was purified and concentrated with 0.45× Ampure PB beads (PacBio, Cat. #100-265-900) for size selection using the BluePippin system (Sage Science, Beverly, MA; Cat #BLF7510) to collect fragments greater than 9 kb. The 15–20 kb average HiFi SMRTbell library was sequenced at UC Davis DNA Technologies Core (Davis, CA) using three 8M SMRT cells, Sequel II sequencing chemistry 2.0, and 30-h movies each on a PacBio Sequel II sequencer.

#### Omni-C Library Preparation

The Omni-C library was prepared using the Dovetail™ Omni-C™ Kit (Dovetail Genomics, Scotts Valley, CA) according to the manufacturer’s protocol with slight modifications. First, specimen tissue is thoroughly ground with a mortar and pestle while cooled with liquid nitrogen. Subsequently, chromatin was fixed in place in the nucleus. The suspended chromatin solution was then passed through 100 and 40 μm cell strainers to remove large debris. Fixed chromatin was digested under various conditions of DNase I until a suitable fragment length distribution of DNA molecules was obtained. Chromatin ends were repaired and ligated to a biotinylated bridge adapter followed by proximity ligation of adapter containing ends. After proximity ligation, crosslinks were reversed and the DNA purified from proteins. Purified DNA was treated to remove biotin that was not internal to ligated fragments. An NGS library was generated using an NEB Ultra II DNA Library Prep kit (NEB, Ipswich, MA) with an Illumina compatible y-adaptor. Biotin-containing fragments were then captured using streptavidin beads. The post-capture product was split into two replicates prior to PCR enrichment to preserve library complexity with each replicate receiving unique dual indices. The library was sequenced at Vincent J. Coates Genomics Sequencing Lab (Berkeley, CA) on an Illumina NovaSeq platform (Illumina, San Diego, CA) to generate approximately 100 million 2 × 150 bp read pairs per GB of genome size.

### Genome Assembly

#### Nuclear Genome Assembly

We assembled the genome of the black abalone following the California Conservation Genomics Project (CCGP) assembly protocol Version 2.0 (Shaffer et al. 2022; Todd et al. 2022), which uses PacBio HiFi reads and Omni-C data for the generation of high-quality and highly contiguous nuclear genome assemblies.

First, we removed remnant adapter sequences from the PacBio HiFi dataset using HiFiAdapterFilt [Version 1.0] (Sim 2021) and generated the initial diploid assembly with the filtered PacBio reads using HiFiasm [Version 0.15-r327] (Cheng et al. 2022) (see Table 1 for assembly pipeline and relevant software). Next, we identified sequences corresponding to haplotypic duplications and contig overlaps on the primary assembly with purge_dups [Version 1.2.5] (Guan et al. 2020) and transferred them to the alternate assembly. We scaffolded both assemblies using the Omni-C data with SALSA [Version 2.2] (Ghurye et al. 2017, 2019) and closed gaps generated during scaffolding with the PacBio HiFi reads and YAGCloser [commit 20e2769] (https://github.com/merlyescalona/yagcloser).

**Table 1.** Assembly pipeline and software usage

Assembly	Software	Version
Filtering PacBio HiFi adapters	HiFiAdapterFilt https://github.com/sheinasim/HiFiAdapterFilt	Commit 64d1c7b
K-mer counting	Meryl	1
Estimation of genome size and heterozygosity	GenomeScope	2
De novo assembly (contiging)	HiFiasm	0.15-r327
Long read, genome–genome alignment	minimap2	2.16
Remove low-coverage, duplicated contigs	purge_dups	1.2.6
**Scaffolding**		
Omni-C mapping for SALSA	Arima Genomics mapping pipeline https://github.com/ArimaGenomics/mapping_pipeline	Commit 2e74ea4
Omni-C Scaffolding	SALSA	2
Gap closing	YAGCloser https://github.com/merlyescalona/yagcloser	Commit 20e2769
**Omni-C Contact map generation**		
Short-read alignment	bwa	0.7.17-r1188
SAM/BAM processing	samtools	1.11
SAM/BAM filtering	pairtools	0.3.0
Pairs indexing	pairix	0.3.7
Matrix generation	Cooler	0.8.10
Matrix balancing	hicExplorer	3.6
Contact map visualization	HiGlass	2.1.11
	PretextMap	0.1.4
	PretextView	0.1.5
	PretextSnapshot	0.0.3
**Organelle assembly**		
Mitogenome assembly	MitoHiFi	2 Commit c06ed3e
**Genome quality assessment**		
Basic assembly metrics	QUAST	5.0.2
Assembly completeness	BUSCO	5.0.0
	Merqury	1
**Contamination screening**		
Local alignment tool	BLAST+	2.10
General contamination screening	BlobToolKit	2.3.3

Software citations are listed in the text.

The primary assembly was manually curated by generating and analyzing Omni-C contact maps and breaking the assembly where major misassemblies were found. No further joins were made after this step. To generate the contact maps, we aligned the Omni-C data against the corresponding reference with bwa mem [Version 0.7.17-r1188, options -5SP] (Li 2013), identified ligation junctions, and generated Omni-C pairs using pairtools [Version 0.3.0] (Goloborodko et al. 2018). We generated a multi-resolution Omni-C matrix with cooler [Version 0.8.10] (Abdennur and Mirny 2020) and balanced it with hicExplorer [Version 3.6] (Ramírez et al. 2018). We used HiGlass [Version 2.1.11] (Kerpedjiev et al. 2018) and the PretextSuite (https://github.com/wtsi-hpag/PretextView; https://github.com/wtsi-hpag/PretextMap; https://github.com/wtsi-hpag/PretextSnapshot) to visualize the contact maps.

We closed gaps generated during scaffolding with the PacBio HiFi reads and YAGCloser [commit 20e2769] (https://github.com/merlyescalona/yagcloser). We then checked for contamination using the BlobToolKit Framework [Version 2.3.3] (Challis et al. 2020). Finally, we trimmed remnants of sequence adaptors and mitochondrial contamination based on NCBI contamination screening.

#### Mitochondrial Genome Assembly

We assembled the mitochondrial genome of the black abalone from the PacBio HiFi reads using the reference-guided pipeline MitoHiFi (https://github.com/marcelauliano/MitoHiFi) (Allio et al. 2020). The mitochondrial sequence of *Mytilus trossulus* (GU936626.1) was used as the starting reference sequence. After completion of the nuclear genome, we searched for matches of the resulting mitochondrial assembly sequence in the nuclear genome assembly using BLAST+ [Version 2.10] (Camacho et al. 2009) and filtered out contigs and scaffolds from the nuclear genome with a percentage of sequence identity >99% and size smaller than the mitochondrial assembly sequence. We identified potential regions with nuclear mitochondrial DNA (numts) by aligning the assembled mitochondrial genome to the final primary assembly using BLAT [v. 36x9, options -out = blast9] (Kent 2002).

#### Genome Size Estimation and Quality Assessment

We generated k-mer counts (*k* = 21) from the PacBio HiFi reads using meryl [Version 1] (https://github.com/marbl/meryl). The generated k-mer database was then used in GenomeScope2.0 [Version 2.0] (Ranallo-Benavidez et al. 2020) to estimate genome features including genome size, heterozygosity, and repeat content. To obtain general contiguity metrics, we ran QUAST [Version 5.0.2] (Gurevich et al. 2013). To evaluate genome quality and completeness we used BUSCO [Version 5.0.0] (Simão et al. 2015; Seppey et al. 2019) with the metazoa (metazoa_odb10) and the mollusca ortholog databases (mollusca_odb10) which contain 954 and 5295 genes, respectively. Despite being less complete, we included the metazoa database to facilitate comparison with previously assembled genomes from the *Haliotis* genus.

Assessment of base level accuracy (QV) and k-mer completeness was performed using the previously generated meryl database and merqury (Rhie et al. 2020). We further estimated genome assembly accuracy via BUSCO gene set frameshift analysis using the pipeline described in Korlach et al. (2017).

## Results

### Nuclear Assembly

**Figure 2.**
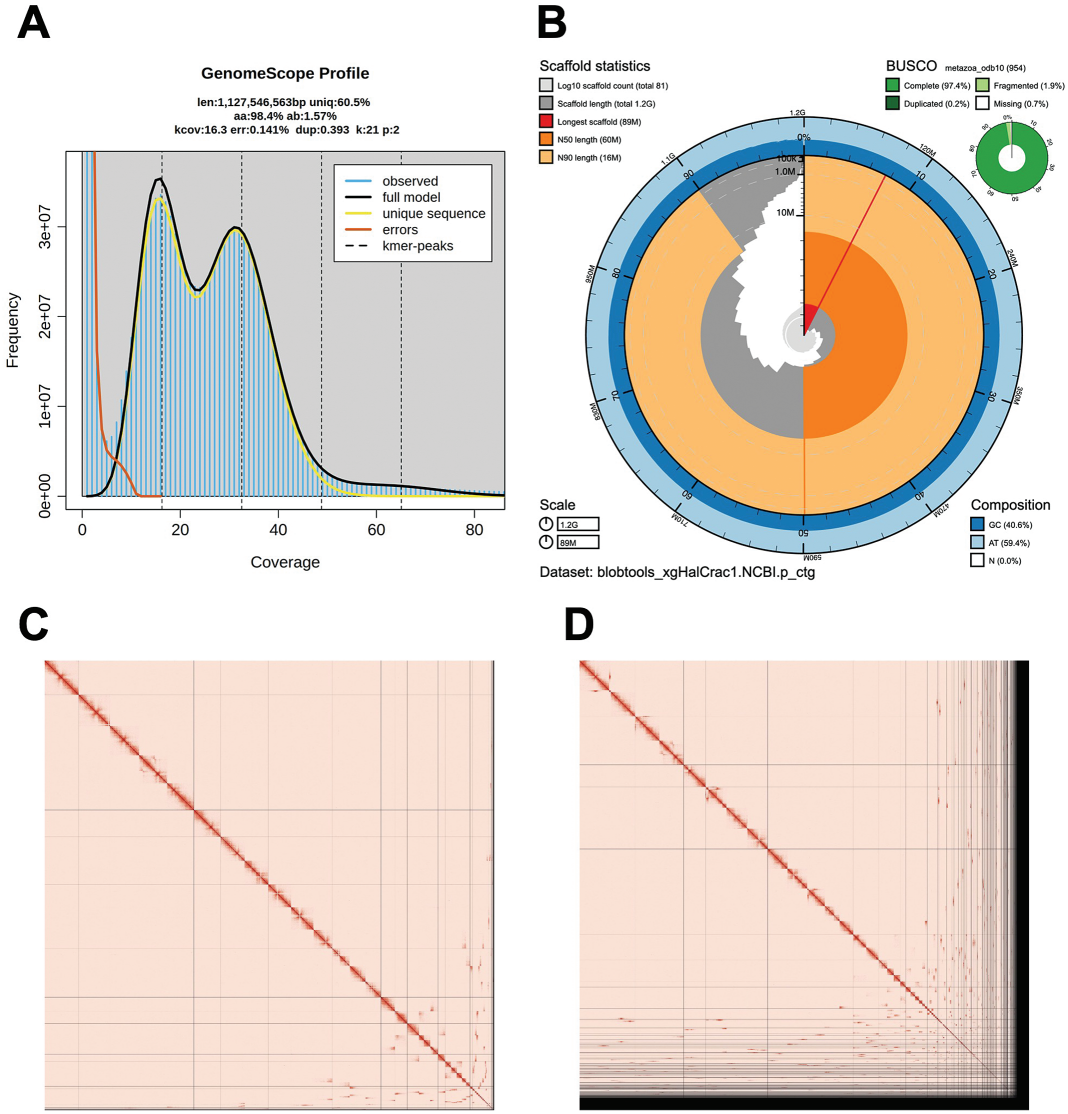
Visual overview of genome assembly metrics. (**A**) K-mer spectra output generated from PacBio HiFi data without adapters using GenomeScope2.0. The bimodal pattern observed corresponds to a diploid genome. K-mers covered at lower coverage but higher frequency correspond to differences between haplotypes, whereas the higher coverage but lower frequency k-mers correspond to the similarities between haplotypes. (**B**) BlobToolKit Snail plot showing a graphical representation of the quality metrics presented in Table 2 for the *Haliotis cracherodii* primary assembly (xgHalCrac1). The plot circle represents the full size of the assembly. From the inside-out, the central plot covers length-related metrics. The red line represents the size of the longest scaffold; all other scaffolds are arranged in size-order moving clockwise around the plot and drawn in gray starting from the outside of the central plot. Dark and light orange arcs show the scaffold N50 and scaffold N90 values. The central light gray spiral shows the cumulative scaffold count with a white line at each order of magnitude. White regions in this area reflect the proportion of Ns in the assembly The dark vs. light blue area around it shows mean, maximum and minimum GC versus AT content at 0.1% intervals (Challis et al. 2020). (**C** and **D**) Hi-C Contact maps for the primary (2C) and alternate (2D) genome assembly generated with PretextSnapshot. Hi-C contact maps translate proximity of genomic regions in 3D space to contiguous linear organization. Each cell in the contact map corresponds to sequencing data supporting the linkage (or join) between two of such regions. Scaffolds are separated by black lines and higher density corresponds to higher levels of fragmentation.

We generated a de novo nuclear genome assembly of the endangered black abalone (xgHalCrac1) using 113.9 million read pairs of Omni-C data and 2.4 million PacBio HiFi reads. The latter yielded ~37-fold coverage (N50 read length 15 971 bp; minimum read length 46 bp; mean read length 15 722 bp; maximum read length of 55 298 bp) based on the Genomescope2.0 genome size estimation of 1.1 Gb. Assembly statistics are reported in tabular and graphical form in Table 2 and Figure 2A,B, respectively.

**Table 2.** Sequencing and assembly statistics, and accession numbers

BioProjects	CCGP NCBI BioProject			PRJNA720569			
	Genera NCBI BioProject			PRJNA765838			
	Species NCBI BioProject			PRJNA777174			
	NCBI BioSample			SAMN22937412			
Genome sequence	NCBI Genome accessions			Primary		Alternate	
	Assembly accession			GCA_022045235.1		GCA_022045225.1	
	Genome sequences			JAJLRC000000000		JAJLRD000000000	
Sequencing data	PacBio HiFi reads	Run		3 PACBIO_SMRT (Sequel II), 2.4M spots, 37.7 G bases, 9.9 Gb			
		Accession		SRR17818992			
	Omni-C Illumina reads	Run		2 Illumina HiSeq X Ten runs: 113.9 M spots, 34.3G bases, 15.8 Gb			
		Accession		SRR17818990-91			
Genome assembly quality metrics	Assembly identifier (Quality code^a^)					xgHalCrac1 (7.7.Q62)	
	HiFi Read coverage^b^			37X			
				**Primary**		**Alternate**	
	Number of contigs			159		1947	
	Contig N50 (bp)			17 462 865		2 050 337	
	Longest contigs			70 066 753		9 187 123	
	Number of scaffolds			81		1215	
	Scaffold N50 (bp)			60 096 789		52 875 726	
	Largest scaffold			89 134 964		82 906 951	
	Size of final assembly (bp)			1 182 252 637		1 201 950 124	
	Gaps per Gbp			52		602	
	Indel QV (Frame shift)			48.66		48.66	
	Base pair QV			62.7732		63.0377	
				Full assembly = 66.00			
	k-mer completeness			80.7192		80.056	
				Full assembly = 99.1857			
	BUSCO completeness (metazoa), *n* = 954		**C**	**S**	**D**	**F**	**M**
		P^c^	97.4 %	97.2%	0.2%	1.9%	0.7%
		A^c^	96.3%	96.3%	0.2%	2.1%	1.4%
	BUSCO completeness (mollusca), *n* = 5,295		**C**	**S**	**D**	**F**	**M**
		P^c^	86.10%	85.20%	0.90%	4.50%	9.40%
		A^c^	85.20%	84.20%	1.00%	4.60%	10.20%
	Organelle	1 complete mitochondrial sequence				CM039063.1	

Assembly quality code x.y.Q derived notation, from (Rhie et al. 2020). *x* = log_10_[contig NG50]; *y* = log_10_[scaffold NG50]; Q = Phred base accuracy QV (Quality value). BUSCO scores. (C)omplete and (S)ingle; (C)omplete and (D)uplicated; (F)ragmented and (M)issing BUSCO genes. *n*, number of BUSCO genes in the set/database. Bp: base pairs.

Read coverage has been calculated based on a genome size of 1.1 Gb.

P(rimary) and (A)lternate assembly values.

The primary assembly consists of 159 scaffolds spanning 1.18 Gb with contig N50 of 17.4 Mb, scaffold N50 of 60 Mb, largest contig of 70 Mb, and largest scaffold of 89 Mb. The Omni-C contact map suggests that the primary assembly is highly contiguous (Figure 2C). Although it is not chromosome-level, we observed in the contact map that the 10 largest scaffolds appear to be chromosome length (Figure 2C). We checked for telomeric repeats (sequence TTAGGGn) in the primary pseudo haplotype, and found those repeats in 8 of the 10 scaffolds identified previously (in either end but not both). As expected, the alternate assembly, which consists of sequence from heterozygous regions, is less contiguous (Figure 2D). Because the primary assembly is not fully phased, we have deposited scaffolds corresponding to the alternate haplotype in addition to the primary assembly.

The final genome size (1.18 Gb) is close to the estimated values from the Genomescope2.0 k-mer spectra and also close to the genome size of species from the same genus (1.80 Gb for *Haliotis discus hannai*, Nam et al. 2017; 1.50 Gb for *Haliotis rufescens*, Masonbrink et al. 2019; 1.71 Gb for *Haliotis laevigata*, Botwright et al. 2019). The k-mer spectrum output shows a bimodal distribution with two major peaks, at ~16 and ~32-fold coverage, where peaks correspond to homozygous and heterozygous states, respectively.

Based on PacBio HiFi reads, we estimated a 0.141% sequencing error rate and 1.56% nucleotide heterozygosity rate. The assembly has a BUSCO completeness score of 97.4% using the metazoa gene set, and a per base quality (QV) of 62. These values are similar to those of *H. rufescens*, the closest *Haliotis* to *H. cracherodii* with its genome assembled (Masonbrink et al. 2019). When using the more complete mollusca gene set, the assembly has a BUSCO completeness score of 86%.

### Mitochondrial Assembly

We assembled a mitochondrial genome with MitoHiFi. Final mitochondrial genome size was 18 391 bp. The base composition of the final assembly version is A = 26.58%, C = 13.51%, G = 24.19%, T = 35.7%, and consists of 22 transfer RNAs and 13 protein coding genes. This assembly was slightly larger than mitochondrial genomes from other members of the Haliotidae, with *H. laevigata* at 16 545 bp (Robinson et al. 2016), *H. d. hannai* at 16 886 bp (Yang et al. 2015), *Haliotis diversicolor* at 16 186–16 266 bp (Xin et al. 2011) and *Haliotis tuberculata tuberculata* at 15 938–16 521 bp (Van Wormhoudt et al. 2011). We also identified 2280 regions that correspond to potential numts. These regions are located in 34 of the 40 largest scaffolds, with scaffold SCAF_11 having the highest number of appearances (327) and size varying from 3 to 243 bp, with an average size of 28 bp (Supplementary Material 1).

## Discussion

The black abalone genome we present here is not only the first reference genome for this species, it is also a highly contiguous and complete draft genome assembled using both long-read and chromosome-scale sequencing data. Combining these different data types provided an assembly more contiguous than previously attempted with short-read data, with an equally low base error rate. Only 75 mollusk genomes are available on NCBI despite it being the second largest animal phylum. Of these, four belong to *Haliotis* species—*Haliotis rubra* (blacklip abalone; Gan et al. 2019), *H. laevigata* (greenlip abalone; Botwright et al. 2019), *H. rufescens* (red abalone; Masonbrink et al. 2019), and *H. d. hannai* (Pacific abalone; Nam et al. 2017). The black abalone genome will be the fifth one assembled from 57 abalone species and the first representing a critically endangered abalone, thus providing a valuable evolutionary and ecological resource.

Fine resolution genomic data will enable us to tackle three questions that need to be urgently addressed for effective management and conservation of black abalone: 1) What is their population structure across their coastal range? 2) Which scenario underlies observed patterns in their genetic diversity (e.g., physiology and life history, bottleneck due to overfishing or WS, ecological barriers)? 3) Can we identify genetic variants associated with individuals less affected by WS? The outcomes of these questions will inform managers working toward recovery actions of black abalone such as translocations from one region to another, outplanting, or captive breeding. For example, local broodstock or restricted translocations may be required for recovery to be successful if populations are highly structured.

One of the six criteria of the National Oceanographic and Atmospheric Administration’s (NOAA) latest ESA Recovery Plan for the black abalone (National Marine Fisheries Service 2019) directly addresses the lack of information on the species’ genetic diversity and pushes toward “developing a plan for assessing genetic structure across the species’ range.” While microsatellite data has been published for black abalone (Gruenthal and Burton 2008; Beldade et al. 2012), whole-genome data will provide finer resolution information on their genetic diversity and connectivity. Future work aligning resequencing data from across their range to the scaffolded high-quality genome presented here should allow for the detection of regions of greatest genomic diversity (Jain et al. 2018; Rice et al. 2020) and for highly accurate structural variant identification (Chaisson et al. 2019), including resilience to WS and other disturbances like warmer water temperature.

Finally, as other iconic and threatened species belonging to the California coastal ecosystem become sequenced, we advocate for a “community genomics” approach examining key players simultaneously. Combining data on primary producers like kelp, top predators like sea otters, and other abalone species, like the more disease-resistant red abalone, will contribute to a more complete understanding of this ecosystem (Raimondi et al. 2015). The black abalone genome will be a useful tool for the monitoring and management of this critically endangered species and its ecosystem, as well as for improving gene annotations in the Haliotidae family and more generally research in mollusk evolution and adaptation.

## Supplementary Material

esac024_suppl_Supplementary_Material_1

## Data Availability

Data generated for this study are available under NCBI BioProject PRJNA777174. Raw sequencing data for sample W230 (NCBI BioSample SAMN22937412) are deposited in the NCBI Short Read Archive (SRA) under SRR17818992 for PacBio HiFi sequencing data and SRR17818990–SRR17818991 for Omni-C Illumina Short read sequencing data. GenBank accessions for both primary and alternate assemblies are GCA GCA_022045235.1 and GCA GCA_022045225.1; and for genome sequences JAJLRC000000000 and JAJLRD000000000. Mitochondrial sequence under GenBank accession number CM039063.1. Assembly scripts and other data for the analyses presented can be found at the following GitHub repository: www.github.com/ccgproject/ccgp_assembly.
